# Genetic variation in the Nr1d1 transcription factor binding site shapes metabolism‐related protein networks associated with cognitive resilience in an Alzheimer's disease mouse reference panel

**DOI:** 10.1002/alz.70896

**Published:** 2025-11-12

**Authors:** Yu Chen, Tamara K. Stevenson, Yiding Cao, Lauren A. Fish, Julia E. Robbins, Gennifer E. Merrihew, Jea Park, Timothy J. Hohman, Michael J. MacCoss, Catherine C. Kaczorowski

**Affiliations:** ^1^ Department of Neurology The University of Michigan Ann Arbor Michigan USA; ^2^ Department of Genome Sciences University of Washington Seattle Washington USA; ^3^ Department of Pharmacology Vanderbilt Memory & Alzheimer's Center Nashville Tennessee USA

**Keywords:** AD‐BXD, Alzheimer's disease, cognitive resilience, DIA proteomics, mitochondria, mouse reference panel, quantitative trait loci

## Abstract

**INTRODUCTION:**

Our previous work established the AD‐BXD mouse panel as an innovative model for studying the genetic complexity and heterogeneity underlying Alzheimer's disease (AD). In this study, we leveraged this model and proteomics approach to identify protein signatures linked to cognitive resilience in AD.

**METHODS:**

We assessed cognitive performance in 6‐month‐old AD‐BXD mice using contextual fear conditioning and calculated a quantitative resilience score. Frontal cortex proteomes were analyzed using data‐independent acquisition mass spectrometry. Protein quantitative trait loci (pQTL) mapping and transcription factor motif analysis were performed.

**RESULTS:**

Cognitive resilience was highly heritable. Of nine pQTL proteins associated with resilience, eight mapped to a shared locus on chromosome 1, forming a genetically regulated module. This module mediates the link between specific SNPs and cognitive outcomes in AD.

**DISCUSSION:**

These findings reveal a protein network underlying resilience to early AD pathology, with Nr1d1 emerging as a key transcriptional regulator.

**Highlights:**

- Quantitative proteomics analysis using the genetically diverse AD‐BXD mouse panel identified nine proteins whose abundances are under genetic control and associated with differences in cognitive response to early‐onset AD mutations.
- Genetic mapping revealed that the abundances of eight of these nine proteins are regulated by the same genomic region on chromosome 1, forming a module that mediates the relationship between specific SNPs and cognitive outcomes in response to AD mutations.
- Integrating proteomic and genomic data suggests that SNP rs46128598, located in the transcription factor binding site of nuclear receptor subfamily 1 group D member 1 (Nr1d1), is a novel effector of metabolic pathways involved in cognitive performance differences in AD mutant carriers.
- This study indicates that targeting the molecular drivers of genetic resilience to AD mutations, including Nr1d1‐mediated proteins (Ak1a1, Gars1, Nudt3, Ogdh, Ptpn11, Iars2, Uba1, Ppt1, and Tmem223), could lead to new therapeutic approaches to delay the onset and/or progression of AD.

## 1 BACKGROUND

Alzheimer's disease (AD) is the most common form of dementia, characterized by amyloid beta (Aβ) plaques and neurofibrillary tangles that cause synaptic dysfunction, neuronal loss, and progressive cognitive decline.^1^, ^2^ Mutations in genes such as amyloid precursor protein (APP) and presenilin 1 and 2 (PSEN1/2) that increase Aβ production cause early‐onset familial AD (FAD), forming the basis of the amyloid cascade hypothesis.^3^ However, despite decades of therapies aimed at Aβ clearance, clinical outcomes have been disappointing. These failures highlight the need for new strategies that not only prevent pathology but also promote cognitive resilience.

While aging is the primary risk factor for AD, genetics accounts for 50% to 80% of susceptibility variance.^4^ Even individuals with pathogenic mutations show variability in onset and progression, suggesting the influence of genetic variants that confer resilience.^5^, ^6^ Recent findings in rare human kindred families carrying the APOE3 Christchurch and RELN‐Colbos variants have provided case reports of three individuals with high pathological burden who have remained cognitively intact well beyond the expected age of symptom onset.^7^, ^8^ Although genome‐wide association studies have identified multiple AD risk loci, including APOE and TREM2,^9^, ^10^, ^11^, ^12^ resilience cases are rare, limiting genome‐wide tests for resilience genes.^13^, ^14^ Moreover, limited access to human brain tissue at early disease stages complicates efforts to distinguish causal from compensatory mechanisms.

Mouse models provide a powerful platform for studying disease mechanisms under controlled conditions with access to presymptomatic stages and the ability to manipulate specific genes. However, conventional AD mouse models are typically maintained on a single genetic background, most often C57BL/6J (B6), which fails to capture the genetic diversity that influences human AD. Studies have shown that genetic background can significantly impact amyloid pathology,^15^ yet relatively few researchers have examined how background affects cognitive phenotypes. To address the limitations of traditional AD mouse models and enable the discovery of genetic modifiers of resilience, we developed the AD‐BXD mouse panel by crossing the well‐characterized 5XFAD transgenic line^16^ with the BXD recombinant inbred reference population.^17^, ^18^ The BXD panel, derived from C57BL/6J (B6) and DBA/2J (D2) strains, segregates for over six million naturally occurring genetic variants and provides a genetically diverse yet fully isogenic background.^17^, ^18^ The resulting F1 AD‐BXD lines of mice harbor the same human AD mutations driven by the 5XFAD transgene but are genetically diverse across the rest of the genome, allowing systematic examination of how genetic background modifies disease expression.^17^, ^19^ This design overcomes key limitations of conventional models by supporting forward genetic approaches and enabling reproducible investigation of gene‐by‐environment and gene‐by‐treatment interactions.

Recent studies suggest that differences among AD‐BXD lines (from susceptible to resilient) in the onset and progression of cognitive deficits result from cell type‐specific variations in gene expression.^17^, ^19^ These transcriptional changes affect protein synthesis, but RNA and protein levels do not always align, particularly in AD.^20^ Therefore, proteomic analysis in AD‐BXD mice is essential to uncover novel protein drivers of resilience. We used an unbiased data‐independent acquisition (DIA) liquid chromatography‐tandem mass spectrometry (LC‐MS/MS) approach^21^ to quantify protein abundances in frontal cortical tissue from 6‐month‐old AD‐BXD mice and their strain‐matched non‐transgenic (Ntg) littermates, to understand early changes in the proteome during AD pathogenesis. The frontal cortex was selected because early‐onset AD patients often exhibit hippocampal atrophy, suggesting cognitive resilience deficits may manifest in nearby cortical regions involved in encoding and long‐term memory.^22^, ^23^ Using this proteomic approach, we quantified cognitive resilience across strains, identified genetically regulated proteins via pQTL mapping, and integrated these findings with transcription factor motif analysis. We discovered a module of metabolism‐associated proteins regulated by a locus on chromosome 1 and identified a candidate single‐nucleotide polymorphism (SNP) within a predicted nuclear receptor subfamily 1 group D member 1 (Nr1d1) binding motif, highlighting a mechanism by which genetic variation modulates proteomic networks and cognitive outcomes in early‐stage AD.

**RESEARCH CONTEXT**

1. **Systematic review**: Although aging is the primary risk factor for AD, genetic factors account for 50% to 80% of disease susceptibility, and even individuals carrying pathogenic mutations show variable onset and progression, suggesting the presence of resilience‐conferring variants. Traditional mouse models maintained on a single genetic background fail to capture this variability, limiting the discovery of genetic modifiers.
2. **Interpretation**: Using DIA proteomics in the genetically diverse AD‐BXD panel, we identified a genetically regulated network of nine proteins associated with cognitive resilience. Integration with genomic data implicates a SNP within an Nr1d1 transcription‐factor binding site as a regulator of metabolic protein networks. Collectively, these findings extend current knowledge by highlighting mitochondrial bioenergetics as a key mechanism and reveal a genetically regulated protein network that underlies cognitive resilience to early Alzheimer's pathology
3. **Future directions**: We will further validate whether Nr1d1‐mediated regulatory pathways can serve as therapeutic targets to promote cognitive resilience in early AD.

## 2 METHODS

### 2.1 Animals

All animal procedures were performed in accordance with the standards of the Association for the Assessment and Accreditation of Laboratory Animal Care and the National Institutes of Health (NIH) *Guide for the Care and Use of Laboratory Animals* and were approved by the respective Institutional Animal Care and Use Committees. For these studies, mice were group‐housed at either the University of Tennessee Health Science Center or The Jackson Laboratory under a standard 12‐h light‐dark cycle with ad libitum access to food and water. The F1 populations of non‐transgenic BXD (Ntg‐BXD) and AD BXD (AD‐BXD) mice used here were generated as previously described.^17^ Briefly, congenic female C57BL/6J mice hemizygous for the dominant 5XFAD transgene (JAX MMRRC Stock No: 34848‐JAX)^16^ were crossed to males from the genetically diverse recombinant inbred BXD (C57BL/6J × DBA/2J) mouse reference panel.^24^ The resulting F1 progeny represents recombinant inbred backcross mice; at any given genomic locus, each mouse harbors either a paternally derived B or D allele from the BXD parent, along with a maternally derived B6‐5XFAD allele. The inheritance of the 5XFAD transgene follows a Mendelian pattern, with approximately half of the F1 progeny carrying the transgene (designated as AD‐BXD) and the other half being non‐transgenic (Ntg‐BXD). Additionally, to produce the F1 populations of AD‐B6 and AD‐D2 mice utilized in this study, male C57BL/6J (B6) or DBA/2J (D2) mice were crossed with female 5XFAD mice maintained on a C57BL/6J background. Transgene carrier status was confirmed through genotyping conducted either by The Jackson Laboratory Transgenic Genotyping Services or by Transnetyx (Memphis, TN, USA).

### 2.2 Behavioral data and tissue collection

**FIGURE 1:**
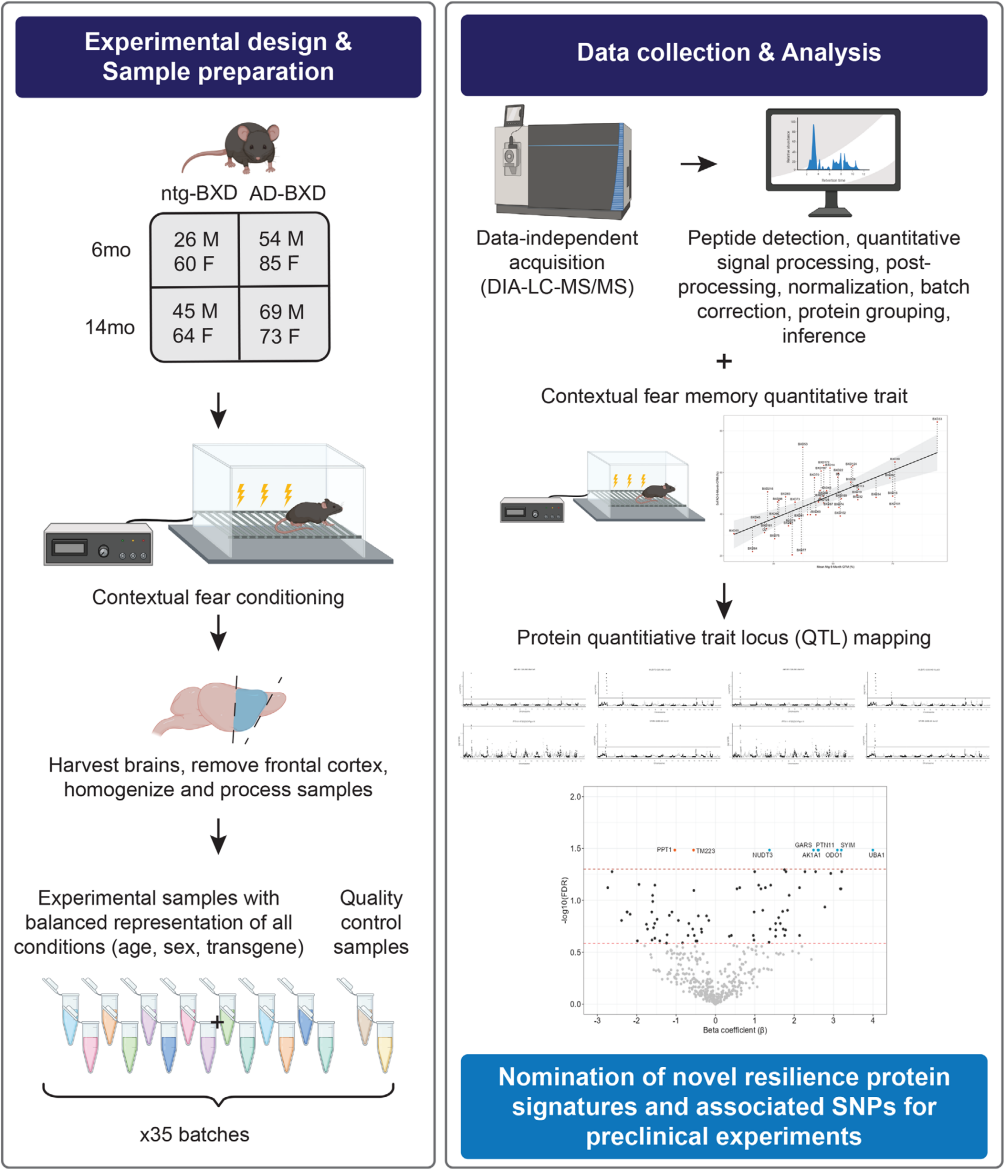
**Frontal cortex DIA LC‐MS/MS proteomic project workflow**. Schematic of the integrated workflow in AD‐BXD mice, combining behavioral assessment, proteomic analysis, and genomic integration, to identify genetic variants associated with cognitive resilience in AD. AD‐BXD mice, carrying high‐risk familial Alzheimer's mutations, and their Ntg‐BXD underwent CFC at 6 and 14 months of age to assess cognitive resilience. Following this assessment, frontal cortical tissue was harvested and prepared for proteomic analysis. The harvested tissue was subjected to DIA‐LC‐MS/MS to measure protein abundance. Subsequent quantitative signal processing involved normalization and batch correction to enhance data reliability and comparability. An analytical method to map cognitive resilience as a continuously varying quantitative trait in AD‐BXD strains was employed. Using computational genomics, nine pQTL‐identified proteins were found to be significantly associated with cognitive resilience, with eight (Akr1a1, Gars, Iars2, Nudt3, Ogdh, Ppt1, Ptpn11, and Uba1) located in the same haplotype block region on chromosome 1. Further integration of proteomic and genomic datasets highlights Nr1d1 as a novel mediator. Its association with a regulatory SNP, rs46128598, suggests potential effects on cognitive resilience. Pathway and network analyses, including KEGG enrichment and protein‐protein interaction networks, were then performed to elucidate the biological mechanisms underpinning these findings. AD, Alzheimer's disease; BXD, BXD recombinant inbred mouse strains; Ntg, non‐transgenic; CFC, contextual fear conditioning; DIA‐LC‐MS/MS, data‐independent acquisition‐liquid chromatography‐tandem mass spectrometry; pQTL, protein quantitative trait loci; AK1A1 [*Akr1a1*], aldo‐keto reductase family 1 member A1; GARS [*Gars1*], Glycyl‐tRNA synthetase; NUDT3 [*Nudt3*], Nudix hydrolase 3; ODO1 [*Ogdh*], oxoglutarate dehydrogenase; PPT1 [*Ppt1*], palmitoyl‐protein thioesterase 1; PTN1 [*Ptpn1*], protein tyrosine phosphatase, non‐receptor type 1; SYIM [*Iars2*], isoleucine–tRNA ligase, mitochondrial; UBA1 [*Uba1*], ubiquitin‐like modifier‐activating enzyme 1; SNP, single‐nucleotide polymorphism; KEGG, Kyoto Encyclopedia of Genes and Genomes.

All mice were trained and tested using a standard contextual fear conditioning (CFC) paradigm, as previously described (Figure 1).^17^, ^24^ Briefly, FreezeFrame software (Coulbourn Instruments, Whitehall Township, PA, USA) was utilized for behavioral assessment. The CFC protocol consisted of a 180‐s baseline period followed by four mild foot shocks (1 s, 0.9 mA), each separated by intervals of 115 ± 20 s. After each shock, a 40‐s post‐shock (PS) interval was established, and the percentage of time spent freezing during each of these intervals was recorded. The percent time freezing during the fourth PS interval (PS4) served as an index of contextual fear acquisition (CFA). Twenty‐four hours after training, mice were returned to the conditioning chamber for a 10‐min testing period, during which the percent time freezing was recorded as an index of contextual fear memory (CFM). All mice underwent CFC testing at 6‐months of age. Immediately after the completion of behavioral testing, tissue from the frontal cortex was harvested and snap‐frozen in liquid nitrogen. Frozen samples were subsequently shipped on dry ice to the University of Washington for proteomic analysis.

### 2.3 Batch design and references

Mouse frontal cortex tissue samples were randomly assigned to batches based on balanced representation across condition group ratios, including genotype, sex, age, and BXD strain (Figure 1).^25^ Each batch contained 14 individual frontal cortex samples and two reference samples. One of these references was derived from a pooled, balanced batch of frontal cortex tissue, serving as a frontal cortex‐specific reference to act as a common calibrant across all batches. This reference was used both to monitor the consistency of the experimental workflow and to correct for variability across different laboratories or experiments.^26^ To further evaluate potential batch effects and ensure consistency in run order, a second reference consisting of a mixture of C57BL/6J (B/B) and DBA/2J (B/D) mouse midbrain and cerebellum tissues from all experimental conditions was included. This additional reference enabled more accurate cross‐comparisons between brain regions in the proteomic profiling.

### 2.4 Tissue homogenization and protein digestion

Frontal cortical tissue was processed using protocols previously established for human brain tissue.^25^ Briefly, snap‐frozen samples were resuspended in 120 µL of SDS lysis buffer, vortexed thoroughly, and sonicated. A 30‐µL aliquot of the resulting lysate – representing one‐quarter of the total volume – was subjected to high‐pressure homogenization at 45,000 psi using a Barocycler 2320EXT (Pressure Biosciences, Canton, MA, USA). After quantification via BCA assay, 50 µg of homogenate was mixed with 800 ng of yeast enolase as a process control to monitor the efficiency of protein digestion. The lysate was then reduced with dithiothreitol and alkylated. Following acidification to facilitate binding to S‐trap columns (Protifi), trypsin was added directly to the column to digest the proteins. Hydrophilic and hydrophobic peptide eluates were subsequently pooled, dried via speed vacuum, and reconstituted in 0.1% formic acid for downstream analysis.

### 2.5 Liquid chromatography and mass spectrometry

As previously described for brain tissue, we used DIA‐LC‐MS/MS for proteomic analysis of mouse frontal cortex tissue (Figure 1).^25^ Each injection sample contained 1 µg of digested peptide, 8 ng of yeast enolase as a protein process control, and 150 femtomoles (fmol) of Pierce Retention Time Calibrant (PRTC) as an internal peptide control.^27^ These samples were analyzed using a Thermo EASY nano‐flow ultra‐high‐performance liquid chromatography system coupled to a Thermo Orbitrap Fusion Lumos mass spectrometer. To ensure data quality, system suitability injections containing 150 fmol of PRTC and bovine serum albumin were run independently and tracked using statistical process control.^28^ Four system suitability runs were performed prior to any sample analysis and repeated after every six to eight sample runs. For chromatogram library generation, six gas‐phase fractionated (GPF) DIA runs with overlapping narrow 4‐m/z isolation windows across a mass range of 400 to 1000 m/z (divided into 100‐m/z intervals) were performed using pooled samples from each batch.^29^ Each individual digested sample was then analyzed using a DIA run with overlapping wide 8‐m/z isolation windows spanning the full 400 to 1000 m/z range. Quality control data were processed and assessed using Skyline and AutoQC software.^28^, ^30^

### 2.6 Quantitative data normalization and batch correction

To reduce residual technical noise, additional processing steps were applied to normalize and correct for batch effects in the quantitative proteomics data (Figure 1). Peptide‐level quantitative data exported from Skyline (Level 2 data) were log_2_‐transformed and median‐normalized to standardize sample intensities to a common scale. A simple linear regression model was then fit, using batch as the predictor variable and peptide abundance as the response, to model and correct for batch‐related bias. The residuals from this model were assumed to represent peptide or protein abundances independent of batch effects. To assess the effectiveness of this correction, principal component analysis (PCA) was performed, projecting the normalized data into reduced dimensions to visualize potential batch‐driven clustering. The resulting normalized and batch‐adjusted peptide abundances were designated as Level 3A data. Subsequently, peptides were grouped into indistinguishable protein clusters and summed to estimate protein‐level abundances, where multiple peptides mapping to the same protein group were merged into single nodes as previously described.^31^, ^32^, ^33^ These processed protein abundance values were designated as Level 3B data.

### 2.7 Data and code availability

All Skyline documents, raw quality control files, and DIA proteomics data are publicly available through Panorama Public. The dataset is registered under ProteomeXchange ID: PXD045403 (DOI: https://doi.org/10.6069/vvb1‐k710) and can be accessed at https://panoramaweb.org/AD‐BXD‐mouse‐PFC‐proteomics.url.^34^ The organization and categorization of the data follow previously published protocols^25^ and are available in five levels of processing. Level 0 includes the raw data in two formats: the native Thermo mass spectrometer format and the demultiplexed mzML format processed via Proteowizard (version 3.0.20064).^35^ Level 1 consists of the zipped Skyline project files. Level 2 contains a CSV file of the integrated peptide peak areas (total area fragments) for each replicate. Level 3A provides batch‐normalized peptide abundances, while Level 3B includes batch‐normalized protein abundance values across all samples.

### 2.8 Quantitative resilience trait

To quantify cognitive resilience, we leveraged the inherent correlation of traits within the BXD panel. This approach enabled direct comparison of CFM performance between AD‐BXD mice carrying the 5XFAD transgene and their genetically matched Ntg‐BXD counterparts lacking the transgene. By comparing animals with and without the AD risk transgene on the same genetic background, we were able to evaluate the specific impact of the transgene on cognitive function, while controlling for background genetic variation.

Specifically, we first calculated the average CFM score for each Ntg‐BXD strain. We then performed a weighted regression of the individual CFM scores from AD‐BXD mice against these Ntg‐BXD strain means. This approach produced a regression line with the same slope and intercept as a regression of strain means, enabling the calculation of individual residuals for each AD‐BXD mouse. These residuals represent the deviation of each transgenic mouse's cognitive performance from the expected strain‐specific baseline. Finally, we standardized these residuals by converting them to *Z*‐scores, which we defined as the quantitative resilience trait.

### 2.9 Heritability estimates

Heritability of the quantitative resilience trait was calculated as previously described,^17^ following established methods for recombinant inbred panels.^36^ Specifically, heritability was estimated using the sum of squares (SS) from an ANOVA model, with BXD strain designated as the independent variable. This calculation was performed for all strains with a sample size of *n* ≥ 2. The proportion of phenotypic variance attributable to genetic differences among strains was computed using the formula below.

$$\;h^{2}=\frac{SS_{\mathrm{strain}}}{SS_{\mathrm{strain}}+SS_{\mathrm{residuals}}}$$

For recombinant inbred panels, measured heritability of a phenotype can be enhanced by considering the strain mean heritability value,^36^ which accounts for multiple measurements within the same strain. In essence, we compared the variance between strains (reflecting genetic diversity, $h^{2}$) and total sample variance (attributable to both genetic and environmental factors, $1\;-\;h^{2})/n$), considering the average number of biological replicates per strain (n). The strain mean heritability formula is as follows:

$$\;h_{ri\bar{x}}^{2}=\frac{h^{2}}{h^{2}+\left(1-h^{2}\right)/n},$$

where n is the average sample size per strain in the experiment.

### 2.10 Protein quantitative trait loci analysis

pQTL analysis was conducted using the same version of GEMMA and genotype files previously employed for mapping the quantitative resilience trait.^37^ Due to limited proteomics coverage across all strains, the analysis was restricted to proteins with expression data available in at least 10 BXD strains. The analysis was performed in *R* (version 4.3.2) using the MatrixEQTL package (version 2.3).^38^

Genotype data were encoded such that homozygous BXD alleles (BB) were assigned a value of 0 and heterozygous alleles (BD) a value of 1. Protein abundance data were log_2_‐transformed and median‐normalized prior to analysis. Only strains with both genotype and proteomic data were included in the final dataset.

The MatrixEQTL engine was configured with a linear additive model (modelLINEAR) and run with a nominal *p* value threshold of 0.05. Multiple testing correction was applied using the Benjamini–Hochberg method to control the false discovery rate (FDR) at <0.05. pQTLs were classified as *cis* when the associated SNP was located within 5 Mb of the gene encoding the protein and *trans* when located more than 5 Mb away or on a different chromosome.^39^ Quality control metrics, including *p* value distributions and histogram outputs, were assessed to validate the robustness of the model. For these proteins, we used their UniProt protein IDs as their identifiers.

### 2.11 Differential expression analysis

Two complementary differential expression analyses of protein abundance were performed using *R* and the limma package.^40^ In the first approach, a linear regression model was applied using the quantitative resilience trait as the predictor variable and normalized protein abundance as the outcome. For each protein, abundance levels were regressed against quantitative resilience trait scores to estimate *β* coefficients representing the strength and direction of association. *P* values for each protein were adjusted for multiple comparisons using the Benjamini–Hochberg method to control the FDR below 0.05. In the second approach, strains were stratified by genotype at a specific allele – either homozygous C57BL/6J (BB) or heterozygous DBA/2J (BD) – and between‐group comparison was performed. Fold changes in protein abundance between BB and BD genotypes were computed, and statistical significance was similarly adjusted for multiple testing.

### 2.12 Principal component and mediation analysis

PCA was used to transform the set of correlated pQTL‐resilient proteins into a new set of uncorrelated variables called principal components, each representing a linear combination of the original protein features.^41^, ^42^ Prior to PCA, protein abundance levels were standardized by converting each protein's values to *Z*‐scores (subtracting the mean and dividing by the standard deviation) to ensure equal weighting across features. The variance in the original protein features explained by each principal component was examined to identify those capturing the most information and then to determine which of the first principal components should be included in subsequent mediation analysis.

Mediation analysis was conducted using a structural equation modeling framework based on the approach described by Baron and Kenny.^43^ The model was implemented in *R* using the lavaan package.^44^ In this framework, a haplotype SNP was modeled as the independent variable, the first principal component (PC1, which explained the highest variance) as the mediator, and the strain‐mean quantitative resilience trait as the outcome. The model included estimation of direct, indirect, and total effects. Standard errors were calculated using maximum likelihood estimation with missing data handled by full information maximum likelihood (missing = “ML”). Bootstrap resampling with 1000 iterations was used to compute bias‐corrected and accelerated confidence intervals for the indirect effect. Statistical significance was assessed at a threshold of *p* < 0.05.

### 2.13 Protein‐protein interaction (PPI) network and KEGG pathway analysis

Differential expression analysis was performed on protein abundance data derived from DIA‐LC‐MS/MS using linear modeling approaches implemented in the limma package. Proteins with an adjusted *p* < 0.05 were considered significantly differentially expressed and selected for further pathway and network analysis. To investigate potential functional relationships among these proteins, a PPI network was constructed using data from the STRING database (version 12.0).^45^ A minimum confidence score cut‐off of 0.7 was applied to retain only high‐confidence interactions. The resulting network was visualized and analyzed using Cytoscape software (version 3.10.1).^46^ Network‐level metrics, including degree centrality, betweenness centrality, and clustering coefficient, were calculated to identify hub proteins and key structural features of the interaction map.

Kyoto Encyclopedia of Genes and Genomes (KEGG) pathway enrichment analysis was also performed using the STRING database online platform. The list of differentially expressed proteins was submitted directly through the STRING interface, which applies a hypergeometric test to assess enrichment. Multiple hypothesis testing was corrected using the Benjamini–Hochberg procedure, and pathways with an adjusted *p* < 0.05 were considered significantly enriched.

## 3 RESULTS

### 3.1 Nine proteins identified from pQTL mapping are significantly associated with the heritable cognitive resilience trait in 6‐month‐old female AD‐BXD mice

Previous work defined cognitive resilience as a continuously varying quantitative trait and determined that genetic factors – other than the 5XFAD gene – are responsible for approximately 50% of the variance in cognitive resilience to AD.^47^ A similar approach was taken here to identify genetic factors and protein networks contributing to earlier stages of cognitive resilience that could be observed as early as 6 months, in female AD‐BXD mice (49 strains; two to 17 mice per strain). First, CFM was measured across the Ntg‐BXD control and AD‐BXD experimental mice as percent time freezing on 2 days of a CFC behavioral task^17^ (Figure 1). Initial results showed that CFM scores varied by genetic background in 6‐month‐old female AD‐BXD mice (Figure S1).

CFC depends on coordinated activity across hippocampal, amygdala, and frontal cortical circuits to consolidate contextual fear memories.^48^, ^49^, ^50^ Thus, measuring freezing behavior in this task provides an indirect but established readout of prefrontal involvement in learning and memory.

**FIGURE 2:**
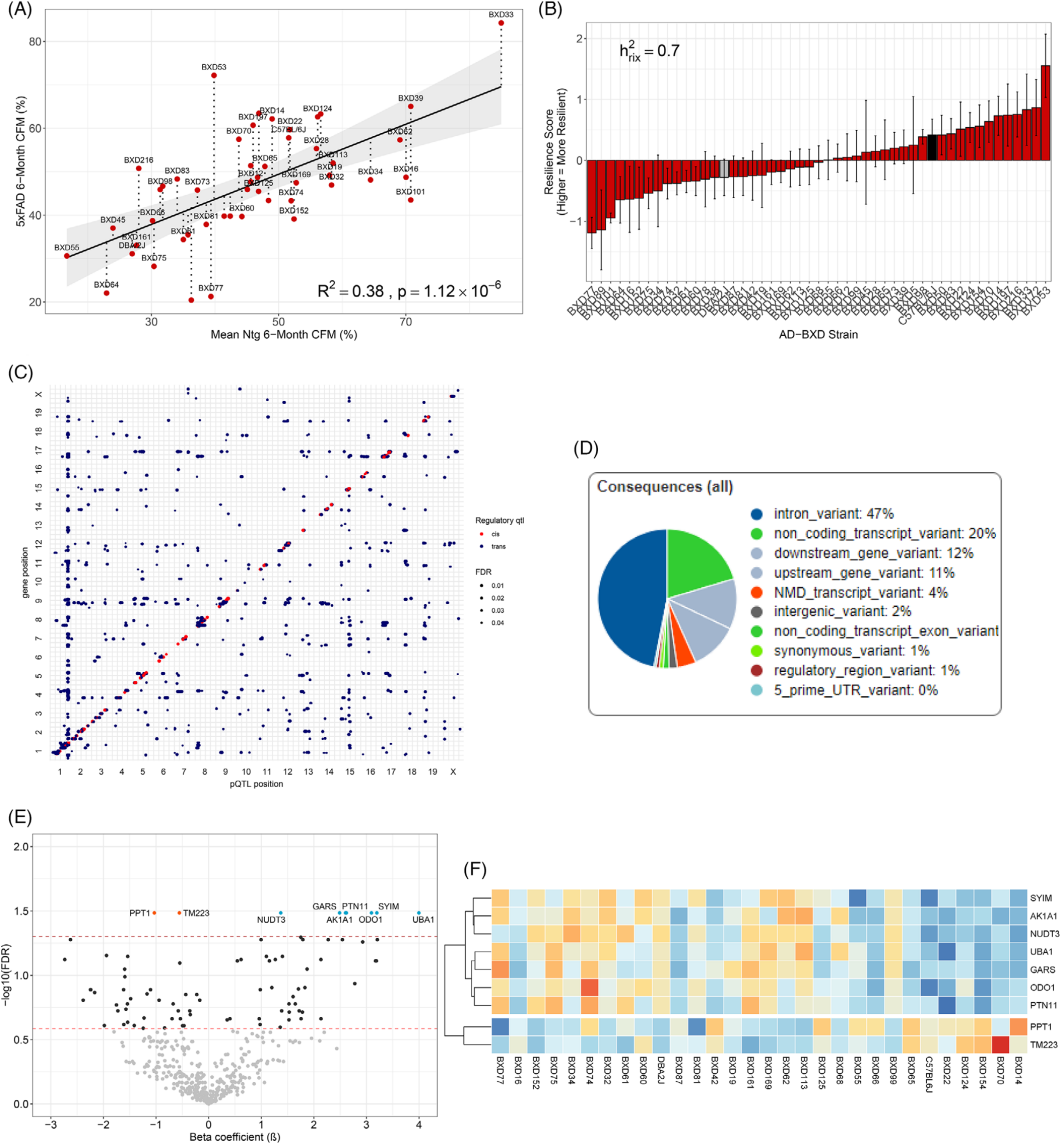
**Nine proteins identified from protein quantitative trait loci mapping are significantly associated with the heritable cognitive resilience trait**. (A) Linear regression analysis of CFM scores between AD‐BXD strains and their respective Ntg‐BXD controls, showing a best‐fit line with *R* ^2^ = 0.38 (*p* = 1.12e^−06^). The analysis confirms that the 5XFAD transgene reduces cognitive function but does not account for all variance in cognitive resilience/susceptibility. (B) Quantitative resilience trait standardized residuals across 49 AD‐BXD strains. The quantitative resilience trait is highly heritable (*h* ^2^ = 0.26, *h* ^2^_rix_ = 0.71), with approximately 71% variance in cognitive resilience due to genetic factors. (C) A pQTL analysis was performed to identify frontal cortical proteins under genetic control. pQTL analysis identified 442 significant proteins (FDR < 0.05) with pQTL peaks; 18% were cis‐pQTLs (*red circles*) and 82% were trans‐pQTLs (*blue circles*). SNP localization includes intronic regions (47%), non‐coding transcripts (20%), and gene‐adjacent regions (23%). (D) SNP distribution from the 442 pQTL peaks includes no missense SNPs in exon coding regions. SNP localization includes intronic regions (47%), non‐coding transcripts (20%), and gene‐adjacent regions (23%). (E) Nine proteins significantly associated with the quantitative resilience trait (FDR < 0.05) are depicted: seven positively (*β* > 0) and two negatively (*β* < 0). Proteins positively associated include AK1A1 [*Akr1a1*], GARS [*Gars1*], NUDT3 [*Nudt3*], ODO1 [*Ogdh*], PTN1 [*Ptpn1*], SYIM [*Iars2*], and UBA1 [*Uba1*]. Negatively associated proteins are PPT1 [*Ppt1*]and TM223 [*Tmem223*]. (F) Heat map of *Z*‐score‐normalized abundance levels of the nine significant proteins in AD‐BXD strains. Strains with *β* coefficient values boxed in blue are cognitively resilient, while those in orange are cognitively susceptible. AD, Alzheimer's disease; Akr1a1, aldo‐keto reductase family 1 member A1; BXD, BXD recombinant inbred mouse strains; CFM, contextual fear memory; DIA‐LC‐MS/MS, data‐independent acquisition‐liquid chromatography/mass spectrometry; FDR, false discovery rate; Gars1, Glycyl‐tRNA synthetase; mo, months old; MS, mass spectrometry; Ntg, non‐transgenic; Nudt3, Nudix hydrolase 3; Ogdh, oxoglutarate dehydrogenase; pQTL, protein quantitative trait locus; Ppt1, palmitoyl‐protein thioesterase 1; Ptpn1, Protein tyrosine phosphatase, non‐receptor type 1; SNP, single‐nucleotide polymorphism; Iars2, Isoleucine–tRNA ligase, mitochondrial; Tmem223, transmembrane protein 223; Uba1, ubiquitin‐like modifier‐activating enzyme 1.

The cognitive resilience metric was calculated from the mean CFM (i.e., percent time freezing) score for a given AD‐BXD strain plotted against the mean CFM score for its respective Ntg‐BXD control. A linear regression analysis of these data returned a best‐fit line with *R* ^2^ = 0.38 (*p* = 1.12e‐06; Figure 2A). Overall, the 5XFAD transgene decreases cognitive function in a 6‐month‐old female AD‐BXD mice but does not explain all the variance in cognitive resilience/susceptibility in the 5XFAD‐BXD panel. To quantify the additional unexplained variance, the standardized residual – or how much a given strain deviates from the linear regression line of best fit – which we refer to as the quantitative resilience trait, was measured. Strains with a more positive quantitative resilience trait demonstrate greater cognitive resilience to AD, while strains with a more negative quantitative resilience trait demonstrate cognitive susceptibility to AD. We found that the quantitative resilience trait varied across the 49 AD‐BXD strains and was highly heritable ($h_{ri\bar{x}}^{2}$ = 0.71; Figure 2B). These data indicate that approximately 71% of the variance in cognitive resilience to the AD gene is due to genetic factors. Moreover, the heterogeneity observed in quantitative resilience trait scores across the AD‐BXD strains is similar to that observed in the human FAD population and serves as a translationally relevant correlate for cognitive performance.^51^

Given the high heritability of the quantitative resilience trait, a pQTL analysis was performed to identify frontal cortical proteins under genetic control involved in maintaining cognition in 6‐month‐old female AD‐BXD mice (*N* = 85, 30 strains). Of the 8688 proteins detected from MS/MS analysis, 442 proteins were identified as having significant (FDR < 0.05, *p* value adjusted by Benjamini–Hochberg method) pQTL peaks associated with their genomic location (Figure 2C). Of the 12,151 SNPs identified in 442 pQTL peaks, 2198 (18%) were in cis‐pQTLs, while 9953 SNPs (82%) were in trans‐pQTLs, and 1178 (10%) of the SNPs were localized to chromosome 1. No missense SNPs in exon coding regions were predicted. The majority of variants were located in intronic regions (47%), non‐coding transcripts (20%), and upstream (11%) or downstream (12%) of genes (Figure 2D). To determine if any of the 442 pQTL proteins were associated with cognitive resilience, a linear regression differential expression analysis was performed between protein abundance levels for those proteins, averaged across all AD‐BXD strains, and the quantitative resilience trait to determine a *β* coefficient. The *β* coefficient for each protein indicates the magnitude and direction of the association between a protein's abundance level and the quantitative resilience trait. Nine proteins were significantly (FDR < 0.05, *p* value adjusted by Benjamini–Hochberg method) associated with the quantitative resilience trait (Figure 2E): Seven had a positive association (*β* > 0: AK1A1 [*Akr1a1*], GARS [*Gars1*], NUDT3 [*Nudt3*], ODO1 [*Ogdh*], PTN1 [*Ptpn1*], SYIM [*Iars2*], UBA1 [*Uba1*]) with the quantitative resilience trait, and two had a negative association (*β* < 0: PPT1 [*Ppt1*] and TM223 [*Tmem223*]) with the quantitative resilience trait. The variability in the *β* coefficient values across the AD‐BXD strains is demonstrated in a heat map of these nine proteins, whose abundance levels were *Z*‐score normalized (Figure 2F).

These pQTL‐resilient proteins appear to be specific to the age‐ and sex‐dependent physiology of 6‐month‐old female AD‐BXD mice. Of the 287 pQTL proteins identified in 14‐month‐old female AD‐BXD frontal cortex by DIA‐LC‐MS/MS, 161 overlapped with those in the 6‐month‐old female dataset, yet none were significantly associated with the quantitative resilience trait. Similarly, 46 of 49 and 201 of 642 pQTL proteins from the 6‐ and 14‐month‐old male AD‐BXD datasets overlapped with the 6‐month‐old female set, respectively, but none showed significant association with the quantitative resilience trait (Figure S2).

### 3.2 Genomic locus on chromosome 1 indirectly mediates the relationship between haplotype SNPs and cognitive resilience

**FIGURE 3:**
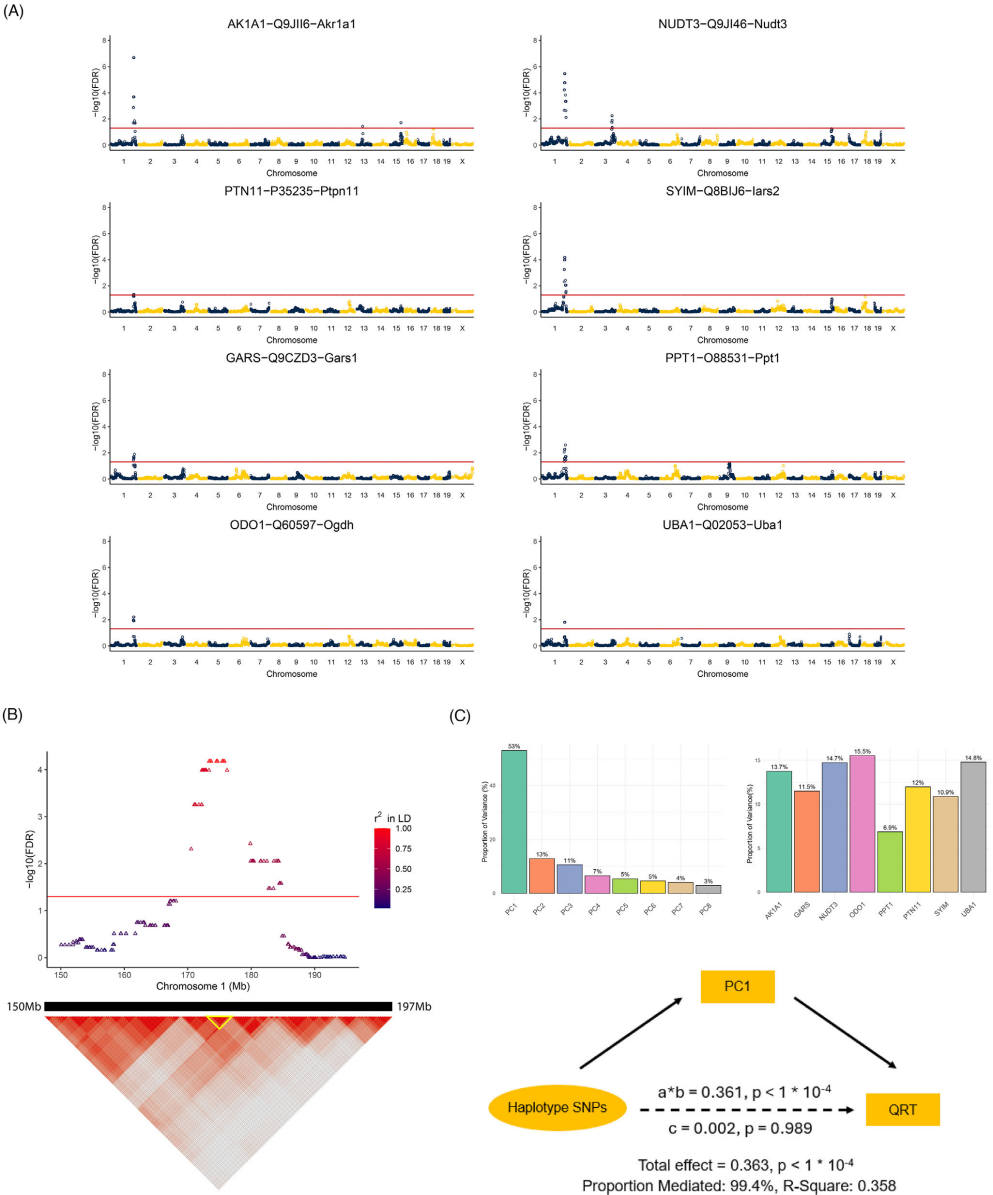
**Genomic locus on chromosome 1 indirectly mediates the relationship between haplotype SNPs and cognitive resilience**. (A) The genomic locus on chromosome 1 hosts eight pQTL proteins AK1A1 [*Akr1a1*], GARS [*Gars1*], NUDT3 [*Nudt3*], ODO1 [*Ogdh*], PTN1 [*Ptpn1*], SYIM [*Iars2*], UBA1 [*Uba1*], and PPT1 [*Ppt1*]), which we term “pQTL resilient proteins,” significantly associated with cognitive resilience. The pQTL for TM223 [*Tmem223*], in contrast, is located on chromosome 19. (B) The genomic region on chromosome 1 (chr1: 173,200,610 to 175,596,722 Mb in GRCm39/mm39 coordinates) harboring 10 significant SNPs (*p* value: 2.11e^−09^, FDR: 6.62e^−05^) is part of a larger haplotype block (chr1: 170.54 to 184.73 Mb). These SNPs, with an *r* ^2^ value of 1.00 (indicating complete non‐random association), are referred to as the haplotype SNPs. (C) To ascertain whether these haplotype SNPs directly or indirectly influence the cognitive phenotype, a mediation analysis was conducted using PCA to address collinearity among the pQTL resilient proteins. PCA transformed the correlated proteins into uncorrelated principal components, enhancing model stability and statistical power. Principal component 1 (PC1, *teal*), encapsulating roughly equal representation of all eight proteins, accounts for 53% of the variance. Mediation analysis using PC1 indicates an indirect effect of 0.361 (*p* < 0.0001) and a negligible direct effect of 0.002 (*p* = 0.989), suggesting that the cognitive phenotype is mediated indirectly through the protein abundance levels rather than being directly influenced by the haplotype SNPs on chromosome 1. AD, Alzheimer's disease; Akr1a1, Aldo‐keto reductase family 1 member A1; BXD, BXD recombinant inbred mouse strains; FDR, false discovery rate; Gars1, Glycyl‐tRNA synthetase; Nudt3, Nudix hydrolase 3; Ogdh, oxoglutarate dehydrogenase; PCA, principal component analysis; PC1, Principal component 1; pQTL, protein quantitative trait loci; Ppt1, palmitoyl‐protein thioesterase 1; Ptpn1, protein tyrosine phosphatase, non‐receptor type 1; SNP, single‐nucleotide polymorphism; Iars2, isoleucine–tRNA ligase, mitochondrial; Tmem223, transmembrane protein 223; Uba1, ubiquitin‐like modifier‐activating enzyme 1.

Eight of the nine pQTL proteins (Akr1a1, Gars1, Nudt3, Ogdh, Ppt1, Ptpn1, Iars2, and Uba1) significantly associated with cognitive resilience are located at the same locus on chromosome 1 (Figure 3A). We have termed these eight proteins “pQTL resilient proteins.” This locus (chr1: 173,200,610 to 175,596,722 Mb in GRCm39/mm39 coordinates) has 10 significant SNPs (*p* value: 2.11e^−09^, FDR: 6.62e^−05^) and the pQTL locus for Tmem223 appears to be part of a larger haplotype block (chr1: 170.54 to 184.73 Mb); Figure 3B. An *r* ^2^ value was calculated to measure the strength of linkage disequilibrium. SNPs with an *r* ^2^ value of 1.00 (*n* = 10; Table S1), indicating complete non‐random association, are here referred to as the haplotype SNPs.

To determine if these haplotype SNPs were directly or indirectly affecting the quantitative resilience trait in 6‐month‐old female AD‐BXD mice, a mediation analysis was performed. A mediation test is based on multiple linear regression analyses and assumes non‐collinearity among independent variables; pQTL resilient proteins, however, were found to be correlated (Table S2). To overcome this collinearity problem, we performed a PCA to transform the correlated pQTL‐identified resilient proteins into uncorrelated principal components. This approach reduces the dimensionality of the variables, produces more stable coefficient estimates, and improves the statistical power of the regression model. PC1, when all eight pQTL resilient proteins are approximately equally represented in the module, was found to explain 53% of the variance (Figure 3C). As PC1 captures the majority of the variability in protein abundance, we subsequently performed a mediation analysis to test if PC1 mediated the quantitative resilience trait. Indeed, we found an indirect mediation effect of 0.361 (*p* < 1e^−04^), with a direct effect of 0.002 (*p* = 0.989). These data indicate that the quantitative resilience trait is not directly caused by the haplotype SNPs on chromosome 1 but indirectly through the high abundance levels of the pQTL‐identified resilient proteins in PC1.

### 3.3 Integrating proteomic dataset with computational genomic data implicates Nr1d1 as a biologically relevant effector of cognitive resilience‐associated proteins

**FIGURE 4:**
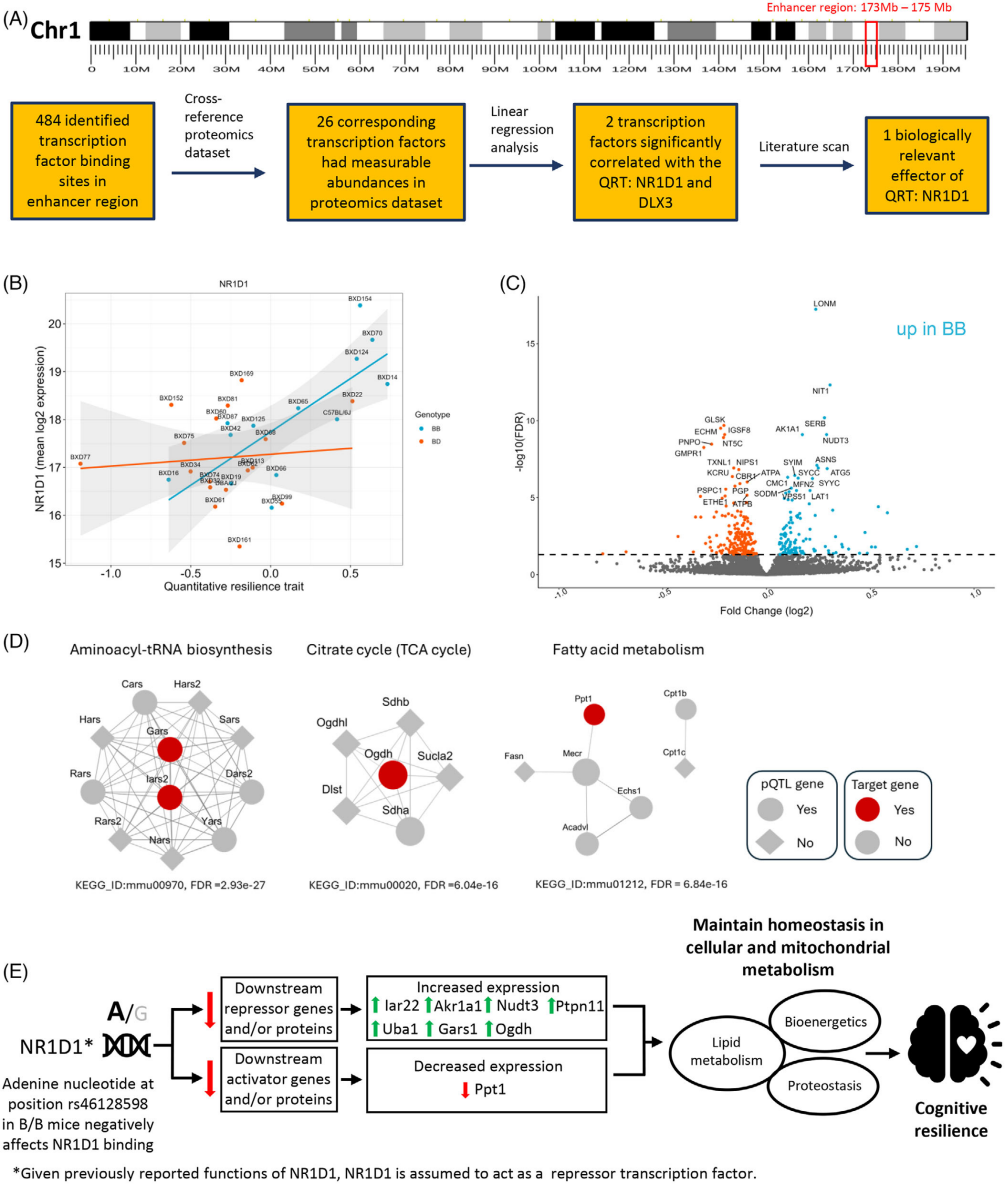
**Integrating proteomics dataset with computational genomic data implicates Nr1d1 as a biologically relevant effector protein of cognitive resilience**. (A) Chromosome 1 (chr1: 173,200,610 to 175,596,722 Mb in GRCm39/mm39 coordinates) was identified as an enhancer region in mice from the ReMap Atlas, with 484 transcription factor binding sites annotated in the JASPAR database. The transcription factors proposed to bind to these 484 binding sites were cross‐referenced with the frontal cortical proteomics dataset. Twenty‐six transcription factors with measurable abundance were detected. (B) Of 26 transcription factors analyzed, Nr1d1 (*r* = 0.546, *p* = 0.002) and Dlx3 (*r* = 0.390, *p* = 0.0034) significantly associate with the quantitative resilience trait, though Nr1d1 shows a stronger correlation. Strains homozygous for C57BL/6J (B/B) at the Nr1d1 allele are more resilient, while heterozygous strains (B/D) are more susceptible to cognitive decline (depicted in blue and orange circles, respectively. (C) Differential expression analysis identifies proteins potentially regulated by Nr1d1 in B/B genotypes. Overall, 309 proteins were found to be significantly differentially expressed; 120 proteins were upregulated and 189 were downregulated. (D) Pathways involving pQTL‐resilient proteins include aminoacyl‐tRNA biosynthesis, TCA cycle, and fatty‐acid metabolism, all of which were previously been implicated in AD pathogenesis. (E) The proposed model suggests the SNP rs46128598 at the Nr1d1 binding site influences cognitive resilience. The adenine nucleotide may affect Nr1d1's binding, potentially leading to changes in the expression of key proteins related to metabolism and energy homeostasis, thereby contributing to cognitive resilience. This hypothesized mechanism involves increased expression of SYIM [*Iars2*], AK1A1 [*Akr1a1*], NUDT3 [*Nudt3*], PTN1 [*Ptpn1*], UBA1 [*Uba1*], GARS [*Gars1*], and ODO1 [*Ogdh*] and decreased expression of PPT1 [*Ppt1*] and TM223 [*Tmem223*], emphasizing Nr1d1's role in maintaining homeostasis during AD pathogenesis. AD, Alzheimer's disease; Akr1a1, aldo‐keto reductase family 1 member A1; BXD, BXD recombinant inbred mouse strains; FDR, false discovery rate; Gars1, Glycyl‐tRNA synthetase; JASPAR, a database of transcription factor binding sites; KEGG, Kyoto Encyclopedia of Genes and Genomes; Nr1d1, nuclear receptor subfamily 1 group D member 1; Nudt3, Nudix hydrolase 3; Ogdh, oxoglutarate dehydrogenase; PC1, Principal component 1; pQTL, protein quantitative trait loci; PPI, protein‐protein interaction; Ppt1, palmitoyl‐protein thioesterase 1; Ptpn1, protein tyrosine phosphatase, non‐receptor type 1; SNP, single‐nucleotide polymorphism; Iars2, isoleucine–tRNA ligase, mitochondrial; TCA, tricarboxylic acid; Tmem223, transmembrane protein 223; Uba1, ubiquitin‐like modifier‐activating enzyme 1.

Results from the mediation analysis led us to postulate the existence of an unknown factor (or factors) effectuating the relationship between the haplotype SNPs and PC1. DNA‐protein interaction assay data (e.g., ChIP‐seq, ChIP‐exo, and DAP‐seq) from the ReMap Atlas previously identified the pQTL hotspot on chromosome 1 as an enhancer region in mouse (Figure 4A).^52^ This region was shown to have 484 transcription factor binding sites from the JASPAR database.^53^ Therefore, we specifically hypothesized that a SNP (or SNPs) in a transcription factor binding site was (were) affecting the downstream expression of proteins in PC1. To test this hypothesis, we cross‐referenced the transcription factors proposed to bind to these 484 binding sites with the frontal cortical proteomics dataset. We identified 26 transcription factors with measurable abundance.

Linear regression analysis between each of these 26 transcription factors and the quantitative resilience trait was performed (Figure S3). Only two transcription factors were significantly positively associated with the quantitative resilience trait: Nr1d1 (*r* = 0.546, *p* = 0.002) and distal‐less homeobox protein (Dlx3; *r* = 0.390, *p* = 0.0034); no transcription factors were found to be significantly negatively associated with the quantitative resilience trait. Differentiating between AD‐BXD strains homozygous for C57BL/6J (B/B) or heterozygous for DBA/2J (B/D) at the *Nr1d1* allele (Figure 4B) shows AD‐BXD strains that genotype B/B are more likely to be cognitively resilient (*R* ^2^ = 0.53, *p* = 0.003; *blue circles*), while strains that genotype B/D are more likely to be cognitively susceptible (*R* ^2^ = 0.05, *p* = 0.72; *orange circles*). An association between Nr1d1 protein abundance and the quantitative resilience trait was not observed in 6‐month‐old male AD‐BXD strains (Figure S4). Based on the documented functions of Nr1d1, including regulation of cellular metabolism, energy homeostasis, and amino acid biosynthesis, we nominate Nr1d1 as a biologically relevant effector of cognitive resilience. While we cannot completely exclude the possibility of DLX3 as a relevant transcription factor involved in regulating cognitive resilience‐associated proteins, the current literature suggests DLX3 is involved in normal morphogenesis during development and tissue‐specific differentiation.^54^, ^55^ The specific SNP that resides in the Nr1d1 transcription factor binding site (JASPAR: MA1531.2) was determined to be rs46128598 (C57BL/6J: A, DBA/2J: G; Figure S5).

To identify other proteins that may be coregulated by Nr1d1 in AD‐BXD strains that genotype B/B at the Nr1d1 allele, a differential expression analysis was performed (Figure 4C). In total, 309 proteins were found to be significantly differentially expressed (adjusted *p* < 0.05); 120 proteins were upregulated and 189 were downregulated (Table S3). KEGG pathway analysis revealed that proteins significantly associated with the quantitative resilience trait, along with other differentially expressed proteins, were enriched in aminoacyl‐tRNA biosynthesis (FDR = 2.93e^−27^), tricarboxylic acid (TCA) cycle (FDR = 6.04e^−16^), and fatty acid metabolism (FDR = 6.84e^−16^) (Figure 4D). A protein‐protein interaction network analysis of differentially expressed proteins segregated at the *Nr1d1* allele is shown in Figure S6. These pathways have all been implicated in AD pathogenesis.^56^, ^57^, ^58^ A working model of putative mechanisms for how the rs46128598 SNP can confer cognitive resilience in AD‐BXD that genotype B/B (vs B/D) at the *Nr1d1* allele is depicted in Figure 4E. Briefly, these data suggest that the adenine nucleotide at position rs43128598 affects Nr1d1 binding; previously reported studies described Nr1d1 as a repressor transcription factor. Assuming that the presence of an adenine nucleotide negatively affects Nr1d1 binding, this impaired binding will lead to decreases in downstream repressor genes and/or proteins and decreases in downstream activator genes and/or proteins. The decrease in repressor gene/proteins will lead to increased expression of Iars2, Akr1a1, Nudt3, Ptpn1, Uba1, Gars1, and Ogdh. The decrease in activator genes/proteins will lead to decreased expression of Ppt1 and Tmem223. The increase or decrease in expression of these proteins affects cellular metabolism, energy production and utilization, and amino acid biosynthesis. Maintaining homeostasis in these critical biological functions during AD pathogenesis contributes to cognitive resilience.

## 4 DISCUSSION

In this study, we used quantitative proteomics based on DIA‐MS to profile the frontal cortex proteome of 6‐month‐old female AD‐BXD mice and their Ntg‐BXD littermate controls to identify novel protein signatures linked to cognitive resilience in early AD.^59^ We employed a novel analytical method to map cognitive resilience as a continuously varying quantitative trait in AD‐BXD strains, and using computational genomics, nine pQTL‐identified proteins were found to be significantly associated with cognitive resilience, with eight (Akr1a1, Gars1, Iars2, Nudt3, Ogdh, Ppt1, Ptpn1, and Uba1) located within a shared haplotype block region on chromosome 1. Notably, this haplotype region, known as Qrr1, is highly enriched for quantitative trait loci (QTLs) previously linked to diverse neural and behavioral phenotypes, including motor behavior, escape latency, emotionality, seizure susceptibility, and responses to various pharmacological substances.^60^ Qrr1 also shows strong concordance with the orthologous human Chr1 q21–q23 interval, which harbors genes such as *Rgs2* (anxiety), *Apoa2* (atherosclerosis), and *Kcnj10* (seizure susceptibility).^61^, ^62^, ^63^, ^64^ This cross‐species alignment suggests that the clustering we observe reflects a conserved regulatory architecture, reinforcing the translational relevance of our findings to human cognitive and neurological traits. Together, our results provide additional evidence that Qrr1 is involved in cognitive resilience. A mediation analysis indicated that these proteins collectively modulated the relationship between the haplotype SNPs and cognitive resilience. We further identified Nr1d1 (also known as Rev‐Erbα), a nuclear receptor transcription factor, as a key regulator, with SNP rs46128598 likely affecting its transcriptional factor binding site and downstream expression. Strains with the B/B versus B/D genotype (C57BL/6J: A, DBA/2J: G) at this SNP show a stronger link between Nr1d1 expression and cognitive resilience scores, suggesting that the adenine nucleotide at position rs46128598 may be a critical cognitive resilience‐associated genetic variant. Differential expression analysis between genotypes revealed that resilience‐associated proteins form biologically functionally integrated protein networks tied to AD‐related pathways, networks that overlap with known Nr1d1‐regulated biological processes. Based on these findings, we propose that Nr1d1 and the identified proteins may promote cognitive resilience by regulating pathways related to mitochondria bioenergetics, oxidative stress, inflammation, and protein turnover – processes essential to maintaining neuronal health and synaptic integrity despite AD pathology.

Nr1d1 is a nuclear receptor primarily recognized for its role in connecting circadian rhythms to metabolic and cognitive processes. As a transcriptional repressor, Nr1d1 significantly influences daily oscillations in gene expression by inhibiting core circadian regulators such as BMAL1, thus serving as a critical integrator of the circadian clock and metabolic pathways.^65^ Beyond its role in circadian regulation, Nr1d1 is crucial in metabolic control and immune responses, linking rhythmic gene expression to inflammation and lipid metabolism.^66^, ^67^ Importantly, Nr1d1 is expressed in various brain regions, including the hippocampus, hypothalamus, midbrain, and cerebellum, underscoring its potential relevance to neurological functions.^68^ Although extensively characterized within circadian biology and metabolism, the involvement of NR1D1 in AD remains relatively underexplored. Notably, human studies indicate that there are local regulatory variants (eQTL) controlling the expression of NR1D1 mRNA and that NR1D1 mRNA expression is altered in the dorsolateral prefrontal cortex (DLPFC) of AD patients (https://agora.adknowledgeportal.org/genes/ENSG00000126368/evidence/rna; Accelerating Medicines Partnership ‐ Alzheimer's Disease (AMP‐AD) consortium).^69^ Available data from the AMP‐AD Agora platform further show that NR1D1 is expressed across multiple human brain regions, with relatively high expression in cerebellum, DLPFC, and temporal cortex. However, in the DLPFC, NR1D1 expression shows nominal associations with hallmark cognitive measures of AD pathology (Braak stage, CERAD score, and cognitive diagnosis) (Figure S7).^70^

Animal models have provided further insight into Nr1d1's multifaceted roles in AD pathology. Deletion or inhibition of Nr1d1 in mouse models enhances Aβ clearance by microglia, suggesting Nr1d1 may typically suppress microglial phagocytic activity.^71^ Conversely, pharmacological activation of Nr1d1 has led to improved cognitive function and reduced amyloid accumulation in aging AD mouse models, highlighting a complex, context‐dependent influence.^72^

Recent evidence also implicates Nr1d1 significantly in tau‐related neurodegeneration and neuroinflammation. Conditional microglial deletion of Nr1d1 exacerbates tau pathology, intensifies neuroinflammation, and disrupts microglial lipid metabolism, indicating a protective role against tau‐induced damage.^73^ Furthermore, Nr1d1 negatively regulates autophagy and mitophagy, critical cellular processes for neuronal maintenance and clearance of pathological proteins. Enhancing autophagy through inhibition of Nr1d1 has demonstrated beneficial effects in several experimental AD models.^71^ It is important to acknowledge that the observed effects of Nr1d1 in our study were specific to 6‐month‐old female AD‐BXD mice and were not replicated in older cohorts or male mice (Figure S4). This finding aligns partially with conflicting evidence from other studies and highlights a potential sex‐ and age‐specific influence of Nr1d1 on cognitive resilience. Thus, future studies should systematically examine these variables to comprehensively elucidate Nr1d1's role in AD.

While previous studies of Nr1d1 primarily emphasized its role in linking circadian rhythms to metabolic and cognitive functions, our identification of novel putative downstream targets suggests additional regulatory roles for Nr1d1 in stabilizing cellular energy balance and antioxidant defenses, both of which are metabolic activities highly dependent on mitochondria. Notably, several previously identified resilience‐associated proteins mediated by Nr1d1 have also been implicated in mitochondrial processes, highlighting their relevance to cognitive resilience in AD. Among these proteins, Gars1 and Iars2 are notable for their roles. Both enzymes play essential roles in mitochondrial protein synthesis; Gars1 uniquely localizes to both cytosolic and mitochondrial compartments, while Iars2 is dedicated exclusively to mitochondrial translation.^74^, ^75^ Dysfunction in these enzymes can severely disrupt mitochondrial translation, impairing energy metabolism and leading to increased reactive oxygen species (ROS) generation, both central features in AD pathology.^75^, ^76^

In addition to their roles in mitochondrial translation, some resilience‐associated proteins identified in our study appear to modulate oxidative stress, an important driver of neuronal dysfunction in AD. Akr1a1, a member of the aldo‐keto reductase superfamily, functions in detoxifying reactive aldehydes and ketones generated during oxidative stress. Reactive intermediates such as 4‐hydroxy‐trans‐2‐nonenal can damage proteins and nucleic acids, forming adducts that compromise cellular integrity.^77^ Elevated Akr1a1 abundance may help mitigate these effects by reducing aldehyde burden, thereby preserving cellular function. Likewise, Nudt3 contributes to oxidative stress defense by hydrolyzing nucleoside diphosphate derivatives damaged by ROS, preventing their incorporation into DNA and limiting mutagenesis.^78^, ^79^ Together, the protective actions of Akr1a1 and Nudt3 likely support genomic stability and metabolic homeostasis in the AD brain. Their association with cognitive resilience underscores the relevance of oxidative stress regulation as a potential mechanism of preserved neuronal function in the face of AD pathology. Further, enzymes such as Ogdh and Ppt1, along with Gars1 and Iars2, provide additional links between mitochondrial functionality and AD. OGDH is integral to the mitochondrial TCA cycle. Dysfunction of Ogdh leads to impaired energy metabolism and increased oxidative stress. Pathogenic variants in Ogdh have been shown to reduce protein stability and enzyme activity, resulting in metabolic imbalance and neurodevelopmental abnormalities.^80^, ^81^ Ppt1 (is involved in lysosomal degradation pathways essential for protein homeostasis).^82^ Mutations in Ppt1 lead to neurodegenerative conditions,^83^ underscoring its role in neuronal health and suggesting a potential intersection with AD pathology through compromised mitochondrial function and impaired proteostasis. Importantly, although none of these proteins have been genetically associated with late‐onset AD, many of them – including Gars1, Iars2, Akr1a1, Nudt3, Ogdh, Ppt1, Ptn11, and Tmem223 – exhibited significant RNA‐level expression changes and are regulated by eQTLs (https://agora.adknowledgeportal.org/).^69^

Together, our findings highlight a genetically regulated network of proteins that may underline cognitive resilience to early Alzheimer's pathology, with Nr1d1 emerging as a key transcriptional regulator within this network. We demonstrated that resilience‐associated proteins converge on mitochondrial function, oxidative stress regulation, and cellular homeostasis – biological processes essential for maintaining neuronal integrity in the face of neurodegeneration. The sex‐ and age‐specific effects observed in our study further underscore the importance of stratified approaches in AD research. Future studies are warranted to validate these findings across diverse models and to investigate whether targeting this Nr1d1‐centered molecular axis can inform new therapeutic strategies aimed at promoting cognitive resilience in AD.

## CONFLICT OF INTEREST STATEMENT

Gennifer E. Merrihew and Michael J. MacCoss declare the following competing financial interests: The MacCoss Lab at the University of Washington has a sponsored research agreement with Thermo Fisher Scientific, the manufacturer of the instrumentation used in this research. Michael J. MacCoss is a paid consultant for Thermo Fisher Scientific and Alnylam Pharmaceuticals. The MacCoss Lab also maintains service agreements with Agilent, Bruker, Shimadzu, Thermo Fisher Scientific, and Waters. Catherine C. Kaczorowski serves as an advisory member of the National Institute on Aging (NIA) Reserve and Resilience Non‐Human Studies Workgroup and as a scientific advisor to the Resilience/Resistance Against Alzheimer's Disease in Centenarians and Offspring (RADCO) project. She is an external advisory board member for the Special Mouse Strains Resource (P40OD011102) at The Jackson Laboratory, an advisor to the NIH Alzheimer's Disease Sequencing Project (ADSP), a scientific advisor to the NIA Functional Genomics Consortium, a member of the NIH Cognitive Aging Summit IV Planning Committee, and a member of the Board of Directors of the American Federation for Aging Research (AFAR). Catherine C. Kaczorowski is a co‐inventor on the patent “DLGAP2 as a Therapeutic Target for Alzheimer's Disease and Age‐Related Cognitive Decline” (WO2020092862A1; PCT/US2019/059311; current assignee: The Jackson Laboratory).

Lauren A. Fish is a co‐inventor on patents related to *Neurogenesis* (WO2020214987A1) and *Muscle regeneration and growth* (US20230304012A1), licensed to Bolden Therapeutics. Timothy J. Hohman serves as a consultant for Circular Genomics and as a member of the Scientific Advisory Board of Vivid Genomics. He is also Deputy Editor for *Alzheimer's & Dementia: TRCI* and Senior Associate Editor for *Alzheimer's & Dementia*.

The remaining authors declare no competing interests. All author disclosures are available in the Supporting Information.

## CONSENT STATEMENT

This study involved only animal subjects. All procedures were approved by the Institutional Animal Care and Use Committees and conducted in accordance with relevant guidelines and regulations.

## Supporting information




**Supplementary Figure 1**: Contextual fear memory scores vary by genetic background in 6‐month‐old female AD‐BXD mice.

**Supplementary Figure 2**: Protein quantitative trait loci resilient proteins are unique to 6‐month‐old female AD‐BXD mice.

**Supplementary Figure 3**: Nr1d1 and DLX3 are significantly positively associated with the quantitative resilience trait.

**Supplementary Figure 4**: Nr1d1 protein abundance levels are not associated with the quantitative resilience trait in 6‐month‐old male AD‐BXD mice.

**Supplementary Figure 5**: One unique SNP resides in Nr1d1 transcription factor binding site.

**Supplementary Figure 6**: Protein‐protein interaction network of differentially expressed proteins segregated at the Nr1d1 allele in 6‐month‐old female AD‐BXD mice.

**Supplemental Figure 7**: Human NR1D1 RNA expression evidence from AMP‐AD Agora platform.

**Supplementary Table 1**: Haplotype single‐nucleotide polymorphisms located on chr1: 173,200,610 to 175,596,722 Mb in GRCm39/mm39 coordinates.

Supporting Information
