# Supplementary material for: Genetic variation in the Nr1d1 transcription factor binding site shapes metabolism‐related protein networks associated with cognitive resilience in an Alzheimer's disease mouse reference panel
Source: Alzheimers Dement. 2025 Nov 12;21(11):e70896. doi: 10.1002/alz.70896 (PMC12611882; doi:10.1002/alz.70896)

Supplemental Figure 1. Contextual fear memory scores vary by genetic background in 6-month old, female AD-BXD mice

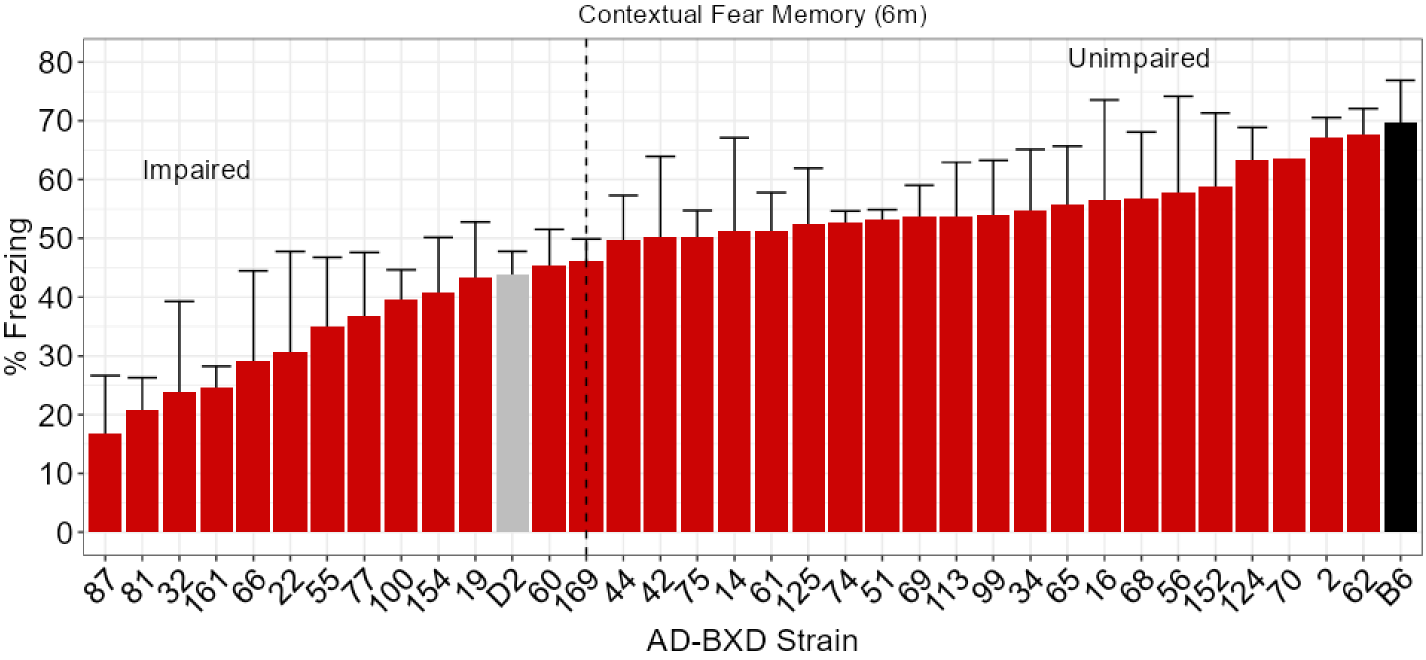

Supplement: Supplementary file 1 — Supplementary Figure 1: Contextual fear memory scores vary by genetic background in 6‐month‐old female AD‐BXD mice. [file ALZ-21-e70896-s003.pdf]
