# Supplementary material for: Genetic variation in the Nr1d1 transcription factor binding site shapes metabolism‐related protein networks associated with cognitive resilience in an Alzheimer's disease mouse reference panel
Source: Alzheimers Dement. 2025 Nov 12;21(11):e70896. doi: 10.1002/alz.70896 (PMC12611882; doi:10.1002/alz.70896)

Supplemental Figure 2. Protein quantitative trail loci resilient proteins are unique to 6-month-old, female AD-BXD mice

Differential Expression with Quantitative Resilience trait

- 14m-Female Subset
- 287 pQTL proteins identified

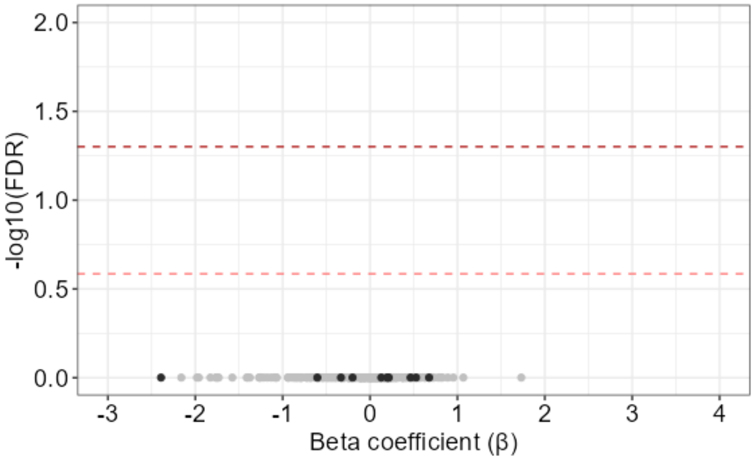

- 6m-Male Subset
- 49 pQTL proteins identified

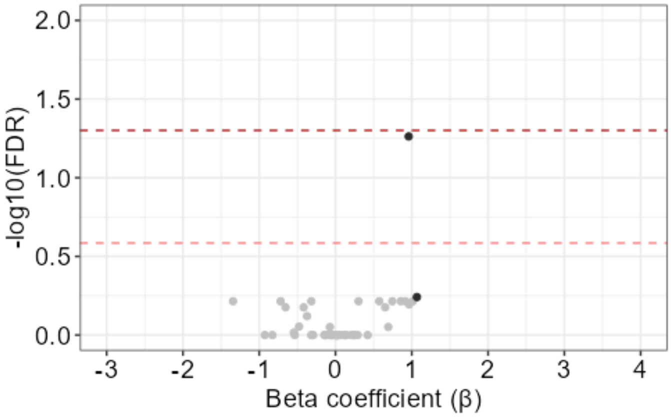

- 14m-Male Subset
- 642 pQTL proteins identified

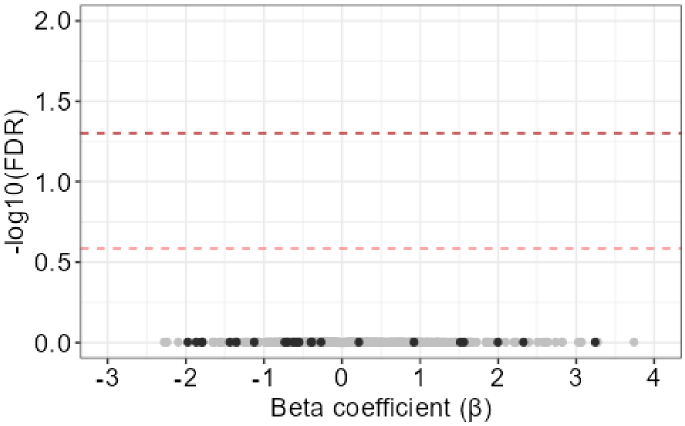

Supplement: Supplementary file 2 — Supplementary Figure 2: Protein quantitative trait loci resilient proteins are unique to 6‐month‐old female AD‐BXD mice. [file ALZ-21-e70896-s009.pdf]
