# Supplementary material for: Genetic variation in the Nr1d1 transcription factor binding site shapes metabolism‐related protein networks associated with cognitive resilience in an Alzheimer's disease mouse reference panel
Source: Alzheimers Dement. 2025 Nov 12;21(11):e70896. doi: 10.1002/alz.70896 (PMC12611882; doi:10.1002/alz.70896)

Supplemental Figure 3. NR1D1 and DLX3 are significantly positively associated with the quantitative resilience trait

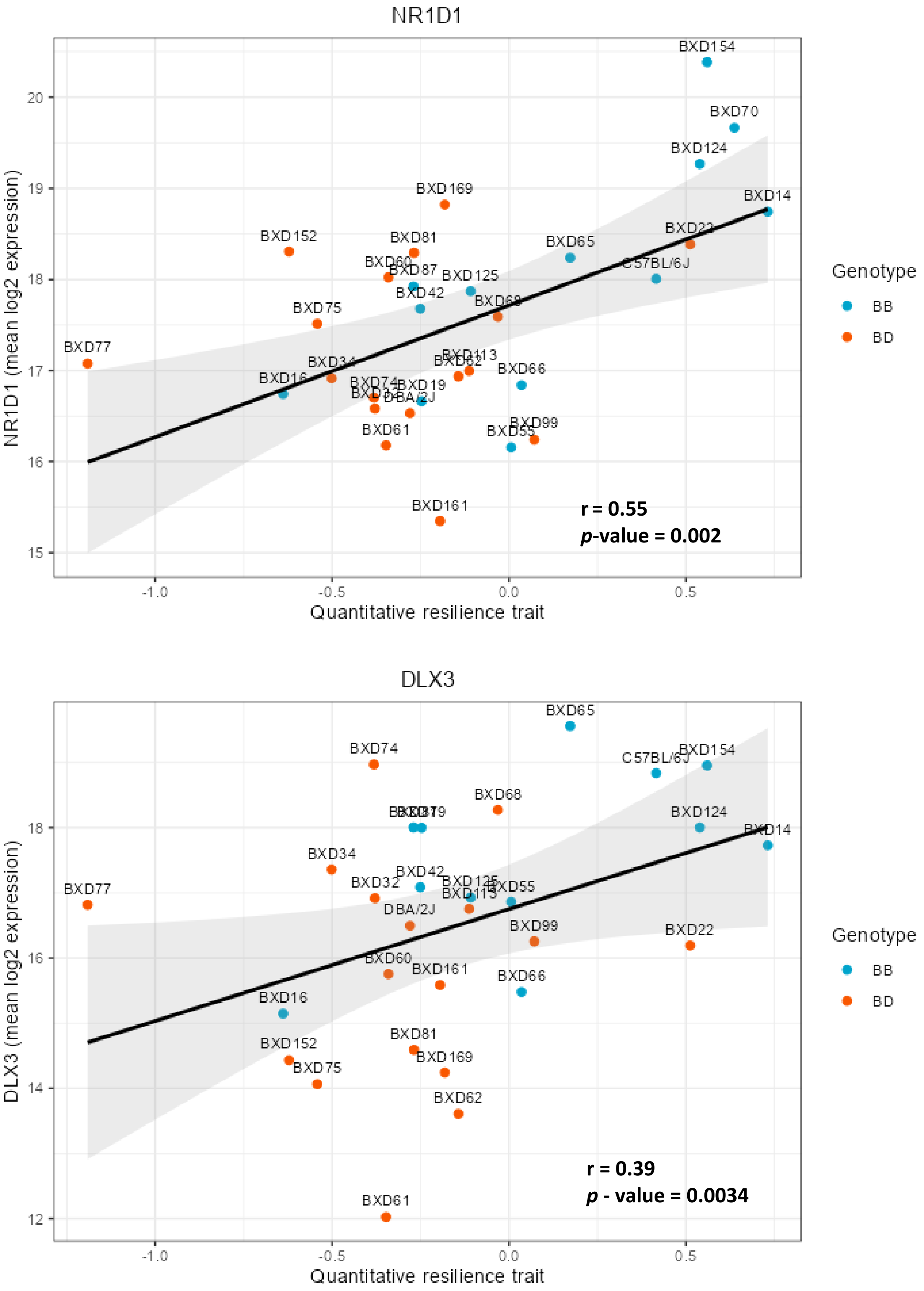

Supplement: Supplementary file 3 — Supplementary Figure 3: Nr1d1 and DLX3 are significantly positively associated with the quantitative resilience trait. [file ALZ-21-e70896-s006.pdf]
