# Supplementary material for: Genetic variation in the Nr1d1 transcription factor binding site shapes metabolism‐related protein networks associated with cognitive resilience in an Alzheimer's disease mouse reference panel
Source: Alzheimers Dement. 2025 Nov 12;21(11):e70896. doi: 10.1002/alz.70896 (PMC12611882; doi:10.1002/alz.70896)

Supplemental Figure 4. NR1D1 protein abundance levels are not associated with the quantitative resilience trait in 6-month-old, male AD-BXD mice

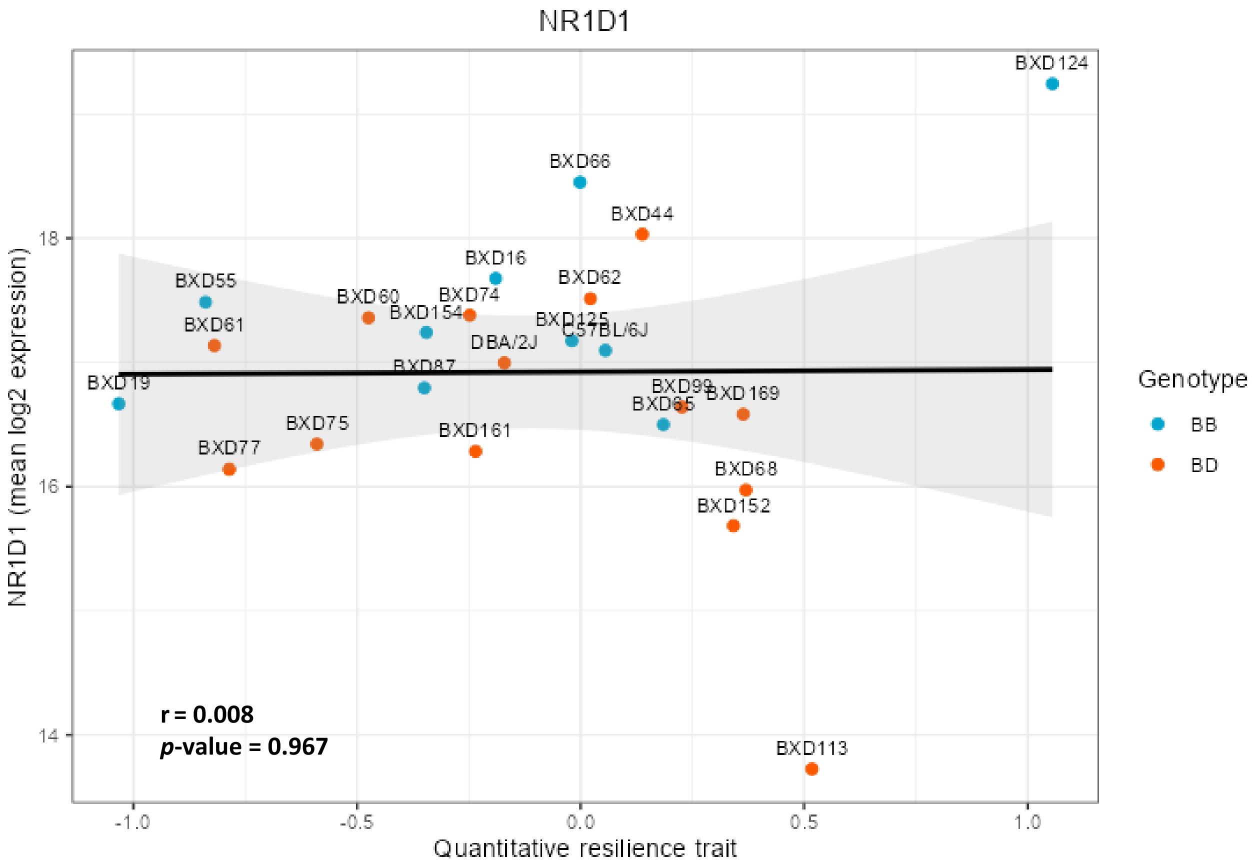

Supplement: Supplementary file 4 — Supplementary Figure 4: Nr1d1 protein abundance levels are not associated with the quantitative resilience trait in 6‐month‐old male AD‐BXD mice. [file ALZ-21-e70896-s004.pdf]
