# Supplementary material for: Genetic variation in the Nr1d1 transcription factor binding site shapes metabolism‐related protein networks associated with cognitive resilience in an Alzheimer's disease mouse reference panel
Source: Alzheimers Dement. 2025 Nov 12;21(11):e70896. doi: 10.1002/alz.70896 (PMC12611882; doi:10.1002/alz.70896)
Supplement: Supplementary file 5 — Supplementary Figure 5: One unique SNP resides in Nr1d1 transcription factor binding site. [file ALZ-21-e70896-s008.pdf]

Supplemental Figure 5. SNP rs46128598 is unique to the NR1D1 transcription factor binding site

Nr1d1 (Nuclear receptor subfamily 1 group D member 1)

Chr	chromStart	chromEnd	TFBS id	Strand	TFname	snps
1	173461137	173461151	MA1531.2	+	Nr1d1	rs46128598

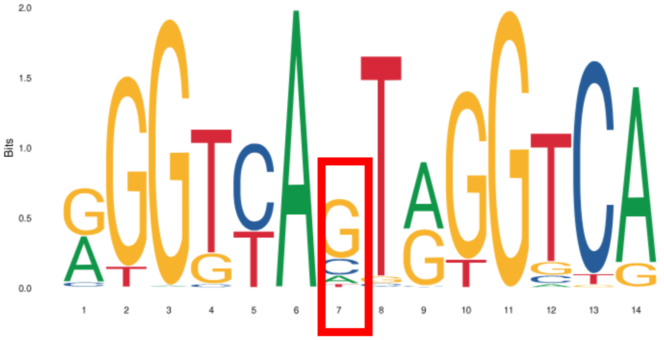

#rs46128598, Chr1: 173461143 (GRCm39), SNP, A/G
C57BL/6J: A
DBA/2J: G

Sequence logo of TFBS motif: MA1531.2
