# Supplementary material for: Genetic variation in the Nr1d1 transcription factor binding site shapes metabolism‐related protein networks associated with cognitive resilience in an Alzheimer's disease mouse reference panel
Source: Alzheimers Dement. 2025 Nov 12;21(11):e70896. doi: 10.1002/alz.70896 (PMC12611882; doi:10.1002/alz.70896)

Supplemental Figure 6. Protein-protein interaction network of differentially expressed proteins segregated at the NR1D1 allele in 6-mo, female AD-BXD mice

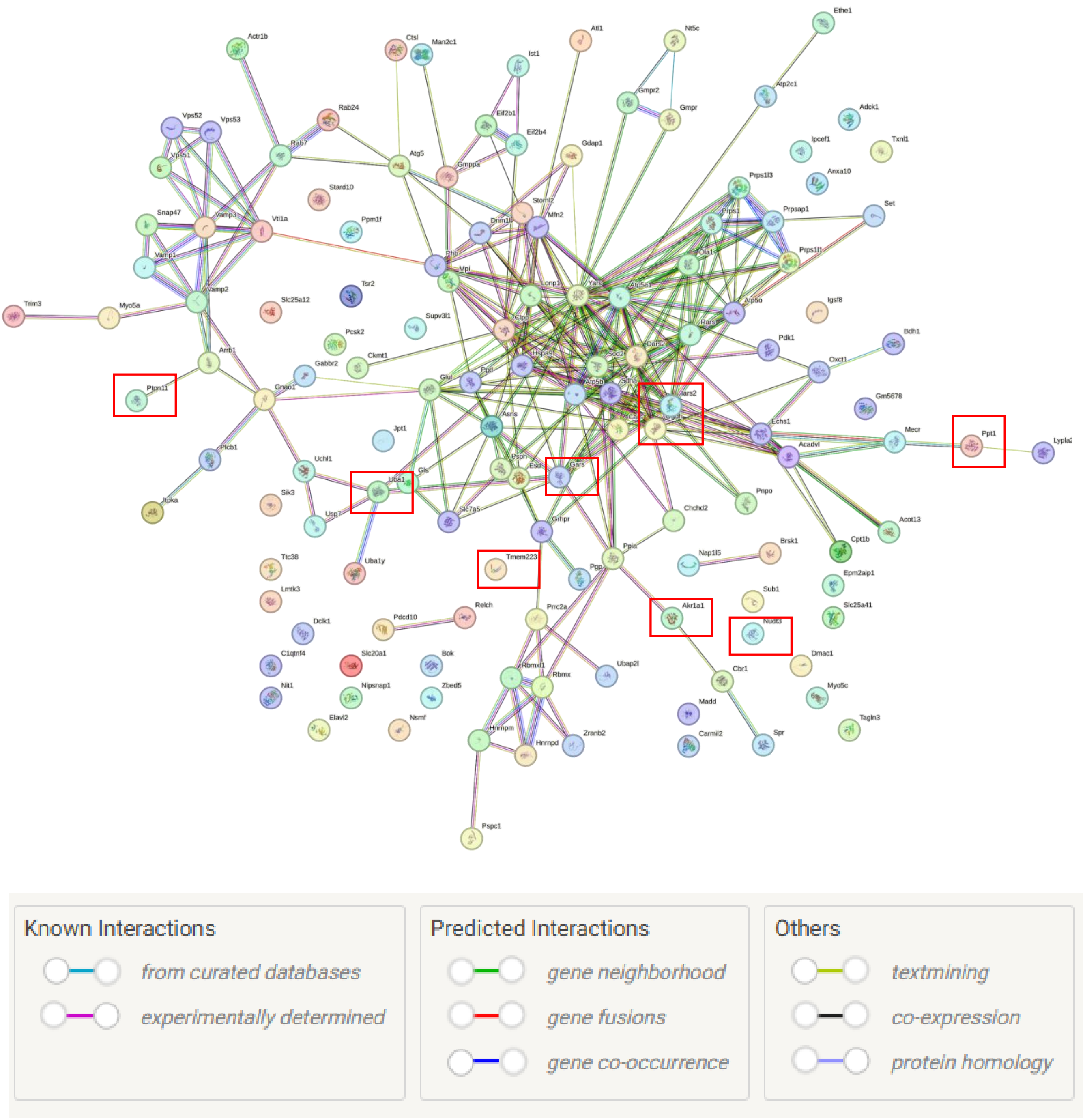

Supplement: Supplementary file 6 — Supplementary Figure 6: Protein‐protein interaction network of differentially expressed proteins segregated at the Nr1d1 allele in 6‐month‐old female AD‐BXD mice. [file ALZ-21-e70896-s001.pdf]
