# Supplementary figures and images for: Genetic variation in the Nr1d1 transcription factor binding site shapes metabolism‐related protein networks associated with cognitive resilience in an Alzheimer's disease mouse reference panel
Source: Alzheimers Dement. 2025 Nov 12;21(11):e70896. doi: 10.1002/alz.70896 (PMC12611882; doi:10.1002/alz.70896)

# Supplemental Figure 7. Human NR1D1 RNA expression evidence from the AMP-AD Agora platform

A

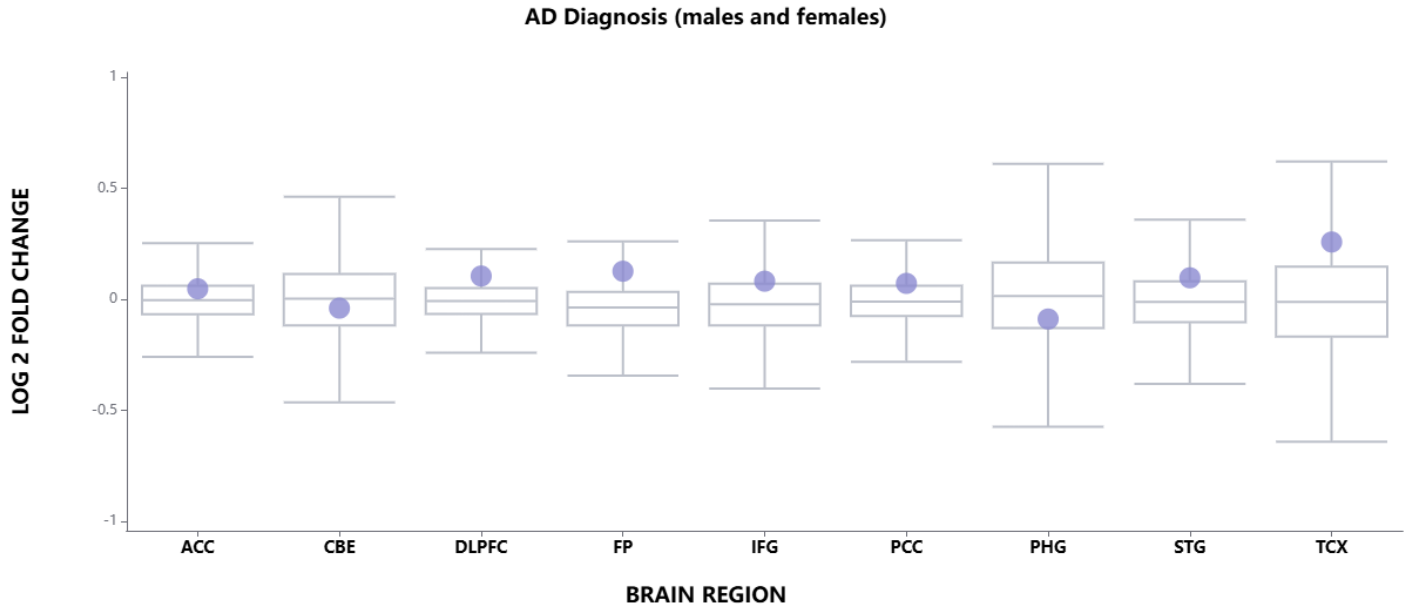

B

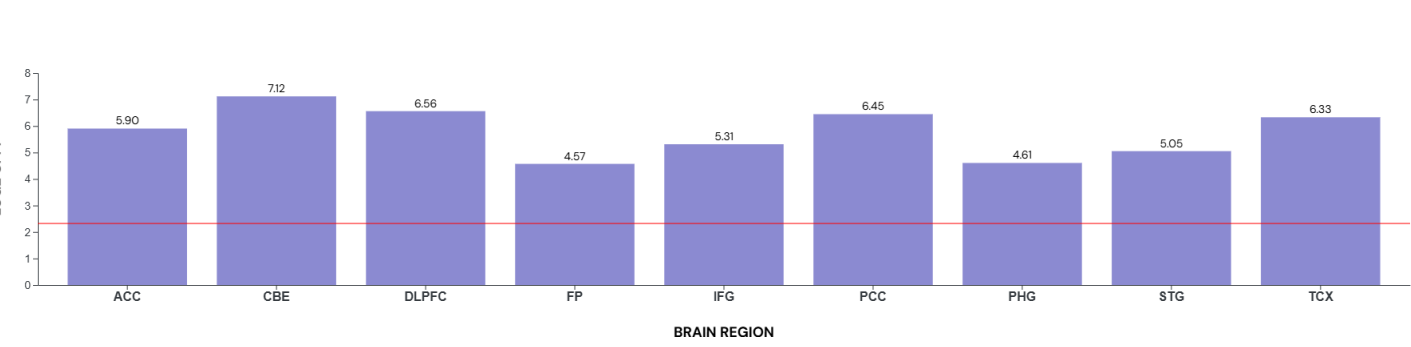

C

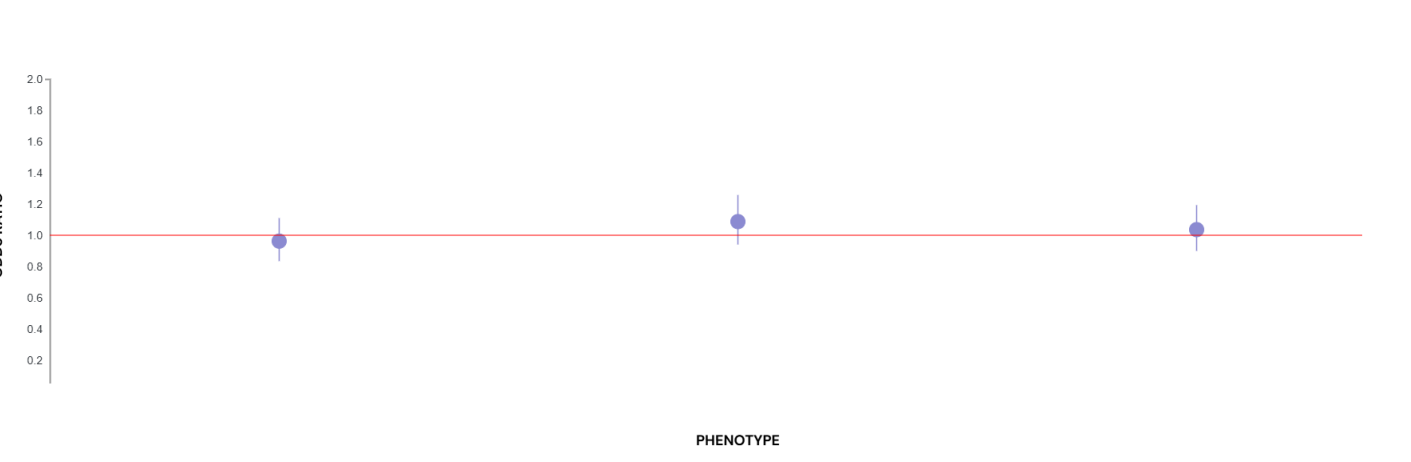

Supplement: Supplementary file 7 — Supplemental Figure 7: Human NR1D1 RNA expression evidence from AMP‐AD Agora platform. [file ALZ-21-e70896-s007.pdf]
